# How are formative assessment methods used in the clinical setting? A qualitative study

**DOI:** 10.5116/ijme.5db3.62e3

**Published:** 2019-11-22

**Authors:** Pernille Andreassen, Bente Malling

**Affiliations:** 1 Centre for Health Sciences Education, Aarhus University, Denmark

**Keywords:** Formative assessment methods, postgraduate medical education, junior doctors, focused ethnography, everyday resistance

## Abstract

**Objectives:**

To explore how formative assessment methods are used and perceived by second-year junior doctors in different clinical settings.

**Methods:**

A focused ethnography study was carried out. Ten second-year junior doctors from different specialties were selected using purposive sampling. The junior doctors were observed during a day in their clinical workplace where formative assessment was in focus. They were subsequently phone interviewed using a semi-structured interview guide regarding their experiences and attitudes towards formative assessment. Field notes from observations and interview transcriptions were analyzed using an inductive content analysis approach, and the concept of “everyday resistance” was used as a theoretical lens.

**Results:**

Three themes were identified: First, there were several barriers to the use of formative assessment methods in the clinical context, including subtle tactics of everyday resistance such as avoidance, deprioritizing, and contesting formative assessment methods.  Secondly, junior doctors made careful selections when arranging a formative assessment. Finally, junior doctors had ambiguous attitudes towards the use of mandatory formative assessment methods and mixed experiences with their educational impact.

**Conclusions:**

This study emphasizes that the use of formative assessment methods in the clinical setting is not a neutral and context-independent exercise, but rather is affected by a myriad of factors such as collegial relations, educational traditions, emotional issues, and subtle forms of resistance. An important implication for the health care sector will be to address these issues for formative assessment methods to be properly implemented in the clinic.

## Introduction

Competency-based medical education (CBME) has become a prevalently recommended approach to post-graduate medical education internationally during the last couple of decades.^1^^-^^5^In CBME, it is essential to be able to measure and document competencies by means of high-quality assessment.^6^^-^^11^ CBME requires summative assessment – or assessment of learning - at the end of training to judge a trainee’s level of competence and assure that competencies have been achieved to a certain standard. 

In recent years, however, formative assessment has become a strong theme in postgraduate medical education as a way to facilitate and enhance learning through-out the training period.^12^^-^^14^Formative assessment - or assessment for learning – aims to identify a trainee’s strengths and weaknesses and to be conducive to progress by means of identifying learning needs and providing feedback in the sense of giving information about the difference between a trainee's current skill level and a given standard.^13^^,^^15^^,^^16^  Although assessment will always have a summative aspect, the use of formative assessment has the potential to provide feedback and give direction for further development. Thus, recommendations from authorities regarding workplace-based assessment goes towards the use of more formative rather than summative assessment in medical education.

A variety of assessment methods, which are used both formatively and summatively, has been developed and validated, including tools of direct observation (e.g. mini clinical evaluation exercise (mini-CEX) and objective structured assessment of technical skills (OSATS)), multisource feedback, retrospective methods (e.g. audit or case-based discussion), and simulations. During or after assessment the supervisor fills in a form and pro-vides feedback.^14^^,^^17^^,^^18^

It is widely agreed upon that formative assessment has great potential for enhancing trainees’ learning.^19^^-^^21^ Apart from improving performance, assessment is also seen as a means to ensure acceptable levels of competence among trainees, so they meet minimum safety standards, and incompetent doctors are identified.^22^  However, in spite of these merits, the implementation of formative assessment has proven difficult.^5^^,^^15^^,^^23^ Indeed, it has been stressed that the “lack of assessment and feedback […] is one of the most serious deficiencies in current medical education practice”.^14^

In Denmark, where the current study takes place, CBME was introduced in 2004. However, a report from the Danish National Health Service in 2012 pointed out that knowledge and use of formative assessment methods were not sufficiently incorporated and not necessarily known by everyone involved in postgraduate medical education,^24^ and this situation does not seem to have changed.

Thus although formative assessment, in theory, is highly suitable for postgraduate medical education, international empirical studies have shown that implementing formative assessment often proves unsuccessful or very difficult.^25^^-^^28^ Since formative assessment takes place in the context of complex clinical practice, it is consequently important to understand how these dynamic processes unfold.^15^  However, to the best of our knowledge, no studies focused on formative assessment in postgraduate medical education have made use of direct observation. The advantage of this approach lies in its ability to uncover actual behaviors and activities, e.g., by studying how people interact and communicate, and how things are organized and prioritized,^29^ hereby offering a more detailed understanding of how assessment is used (or not used). Thus, the aim of this study was to explore how workplace-based assessment methods are actually carried out and perceived in different clinical settings.

## Methods

### Study design

In this study, we made use of a focused ethnography approach. Focused ethnography is a qualitative research approach that has been used to explore ‘fields specific to contemporary society which is socially and culturally highly differentiated and fragmented’.^30^ Unlike traditional ethnography, which is typically characterized by long-term fieldwork and very broad scope, focused ethnography can be described as being problem-focused, concentrating on particular social phenomena, involving predetermined participants, and short-term, episodic participation observation.^31^ However, like traditional ethnography, focused ethnography maintains an inductive approach, including openness to unexpected themes.^32^

### Sampling methods and participants

In order to be able to directly observe how formative assessment methods are being carried out in the clinic, we sampled junior doctors in their second year of specialist training (hereafter referred to as “junior doctors”), who participated in a mandatory seminar on guidance, assessment, and supervision in the fall of 2017.^34^^,^^35^^,^^36^ We chose this way of sampling because the guidance seminar in addition to two conventional seminar days includes a project day that takes place at the junior doctors’ own place of employment. During this project day, the junior doctors are supposed to focus on assessment by preparing, carrying out, and evaluating a clinical performance assessment based on an assessment tool of their own choosing.^36^ By following the junior doctors around their workplace on a project day, where the focus was on formative assessment methods, we were sure that assessment would take place, making it possible to gain insight into how these methods were actually used.

We wanted maximum variation in terms of geographical location and medical specialty and therefore made use of purposive sampling/maximum variation sampling.^33^ After being thoroughly instructed by the first author (an experienced ethnographer) on the sampling criteria, two of the seminar leaders contacted a total of 32 junior doctors who participated in the guidance seminar in order for them to allow the first author to contact them by mail or phone and offer information about the present study. Because the project days took place on ten specific dates, it was only possible to follow ten junior doctors, and therefore the first ten out of the 32 junior doctors to agree to participate ended up being part of the study. Thus, participant observation and interviews with ten junior doctors, six female and four male, were conducted.

### Study settings and data collection

Passive participant observation was carried out on the ten junior doctors’ individual project days at their individual workplace: Three surgical departments, five medical departments, one general practice, and one paraclinical department.^37^ Thus observations took place on ten different days in ten different clinical settings with ten different junior doctors.

On the ten different project days, the first author would follow the junior doctor, typically starting with a morning meeting and then continuing in a range of different activities in the clinic (including operations, ward rounds, consultations, etc.). The first author introduced herself and the purpose of the project to the rest of the ward/colleagues at the morning meeting and introduced herself to colleagues encountered throughout the day, who then verbally consented to the researcher’s presence. Patients who were encountered were notified in advance - typically by a nurse – that an ethnographer was doing a project on the education of doctors, and they were given the opportunity to denounce the researcher’s presence without the researcher being there.

Throughout the project day, the first author would make field note jottings whenever appropriate (typically, while the junior doctor was updating medical charts at the computer), and later wrote up more comprehensive notes about what happened during the day.^38^ Through-out the day, informal and unstructured interviews were carried out with the junior doctors as well as their colleagues (other doctors and nurses). ^39^

The first author conducted formal, semi-structured phone interviews with all ten junior doctors approximately one week after their individual project day. Interviews lasted between 30 and 60 minutes and were based on a semi-structured interview guide that included questions about assessment, overall training, the experience of the project day as well as clarifying questions pertaining to incidents on the specific project day. Six months after the first author carried out short follow-up phone interviews with six of the ten junior doctors. Four did not respond to texts or phone calls.

### Analysis

Field notes and transcriptions of the interviews were read through several times and analyzed following the qualitative content analysis approach.^38^ Initial codes and categories were generated, and initial memos were written and discussed by the authors. After investigating relations between codes, overall themes and patterns were selected and explored in relation to the full data set. Themes and codes were discussed among the authors, before the final analysis was conducted.

The excerpts used in this article were translated from Danish to English by the first author and reviewed by the second author. A fairly large number of interview excerpts have been included in the article as “textual evidence” to further the transparency of the study.^40^

### Using everyday resistance as an analytical lens

Based on the empirical findings, we chose the concept of everyday resistance as a lens for understanding the ways in which formative assessment methods are used in everyday clinical contexts. Everyday resistance has been categorized as being “about how people act in their every-day lives in ways that might undermine power”.^41^ Unlike easily recognizable types of outright resistance such as demonstrations, strikes, or rebellions, everyday resistance is not dramatic or confrontational. Rather it may take place in covert ways and is not politically articulated or formally organized.^41^ It consists of small individual acts of insubordination, e.g. evading or ignoring, manipulating regulations, or doing things slowly or differently than intended by superiors.^42^ These acts are in themselves so small as to not openly contest the dominant, but do altogether create a barrier to power.^42^

Theories of everyday resistance have particularly been used to shed light on how subordinate groups resist domination in subtle ways. Doctors are not usually considered a subordinate group. On the contrary, the title of the doctor is usually linked to a prestigious, authoritative, and superordinate position. However, it is possible for individuals to be simultaneously positioned as powerful and powerless in different systems.^41^ For instance, doctors can be said to be powerful in the doctor-patient relationship, but are at the same time subject to their superiors in the workplace and the rules of the National Health Service.

For our purposes, Michel de Certeau offers a useful approach in his distinction between strategies and tactics. 43Strategies are related to overall power structures and hegemonic discourses deployed by dominant institutions to exert greater control over time, space, and practice, while tactics are individual, daily practices. The latter are acts “in which the weak are seeking to turn the tables on the strong” ^43^, but in ways that are often disguised or ambiguous. In this article, we will argue that the way junior doctors and their senior colleagues use (or do not use) the mandatory formative assessment methods and the associated forms can be seen as tactics of everyday resistance to obligations (strategies) imposed on them from the outside.

### Ethical considerations

The junior doctors received oral and written information about the study and consented to participation both verbally and in writing. Consent to do participant observation in the ten different clinical settings was obtained by email or phone calls to executive consultants in the ten settings. The study was approved by the Danish Data Protection Agency (J. No. 2016-051-000001, Sequential No. 656). The study was exempt from ethical approval from The Central Denmark Regional Committees on Biomedical Research under which this study is classified, because it did not involve medical products, experiments including patients, etc. The American Anthropological Association’s code of ethics was followed.^44^

## Results

In the data analysis, three different themes emerged relating to 1) the actual use of formative assessment methods in the clinic, 2) selections the junior doctors made when arranging an assessment, and 3) ambiguous attitudes towards the use of formal assessment methods.

### Actual use of formative assessment methods in the clinic

Observation revealed a number of conditions in the complex clinical workday, which affected the way formative assessment methods were carried out, as recounted below.

#### Overall busyness in the clinic

During the ten project days, the clinic was clearly characterized by busyness. For instance, at morning report, a frustrated chief physician informed the junior doctor who was trying to introduce the project day and explain the presence of the first author, that “Today is not a day for projects or anthropologists. It’s a force majeure day, where everyone has to take on extra patients” (Chief physician no. 10, medical ward).

Despite the busyness, all ten junior doctors succeeded in carrying out their assessment assignment. However, the six

junior doctors who chose to be assessed by others were challenged in terms of timing and logistics, because it was difficult to get both the assessor and nurse – and sometimes patient - aligned. While all senior doctors who were approached readily agreed to carry out the assessment, in two instances the assessors left after being paged during the consultation and thereby missed large parts of it.

According to informal interviews with junior doctors, clinical care is unsurprisingly the number one priority. Even in clinics where formative assessment was actively encouraged, e.g. by the educational leader, it could be difficult to carry out because of logistics and busyness. In an interview, one junior doctor from a medical ward explained that during a quiet day, the educational leader urged her to have a mini-CEX done. The junior doctor explained:

“We planned it, but I finished off [my rounds] with that patient, […] and then a lot of things happened, such as a heart attack, and the patient I was supposed to receive supervision on ended up being transferred to another department. So not a lot came of it.” (Junior doctor no. 2, medical specialty)

In the above examples, assessment can be seen as being resisted by a tactic of deprioritizing with the overall busyness in the clinic being an acceptable reason for this choice. When a pager goes off, when it is a “force majeure day”, when plans for assessing are disrupted, assessment is skipped or given a low priority.

#### Limited familiarity with and different uses of the assessment methods

Observations on the ten project days also revealed differences in the familiarity with assessment methods. In most cases, the junior doctor had to introduce the assessment method and form to the assessor/assessed, who seemed unfamiliar with it. For instance, one junior doctor from a surgical ward was asked by the assessor to explain the scales: “Is 1 highest or lowest?” (Specialist doctor no. 4, surgical ward)

There were also considerable differences as to how assessments were carried out. Some of the assessors filled in the assessment form, while observing the assessed. Others did so while giving feedback and simultaneously explaining why they gave a certain score. Still, others gave feedback without making any use of the form, in one instance commenting: “I’ll fill this [form] in later and put it in your pigeonhole.” (Specialist doctor no. 2, medical ward)

The feedback the junior doctors gave or received also varied greatly. Most assessors, who were typically specialist doctors, used the form as a starting point for giving specific and constructive feedback. Others gave more unstructured feedback, and some merely announced that “That went well” without elaborating.

Overall, the senior doctors who were asked to assess seemed to have limited familiarity with the different types of assessment methods the junior doctors asked them to perform. None of them declined the junior doctors’ request, but their unfamiliarity with and diverse ways of carrying out the assessment can be seen as a tactic of resistance. By not being acquainted with and by re-articulating assessment and feedback in their own fashion, the senior doctors can be seen as devaluing the importance of the strategically induced formal assessment forms and instead reinforcing their own autonomy, i.e. their own way of doing things.

### Using other types of learning

During the ten project days, many instances of informal supervision occurred where the junior doctors asked others and were themselves asked for advice. This informal supervision was characterized by being ad hoc and unstructured.

In informal interviews, most junior doctors expressed that they were happy with the way that teaching took place in the clinic in ways that did not involve formal assessment methods and forms. For instance, in the general practice, the junior doctor’s main supervisor had a special interest and education in supervision and communication and made use of different teaching models, which the junior doctors found much more useful than the assessment forms, which she found “simplified” (Junior doctor no. 8, general practice).

While some saw the forms as too basic, others experienced them as superfluous. This became especially clear in the paraclinical setting and in the general practice. However, also in one of the surgical wards, a junior doctor repeatedly emphasized that young doctors are constantly being guided and supervised without the use of assessment forms. In the interview, she stated:

“I think that in our specialty, in surgery, at any rate, I just think that we’re really good [at supervising], because we’re always with an older colleague, you can say. Especially during surgery, I always think that, like, when we’re done with a procedure or an operation, well, then you talk it over: What went well, what didn’t go so well, what could you have done differently. And that’s without a form, you can say. So regardless of how many [assessment] forms are filled out, you get something out of it, because we’re constantly in apprenticeship.” (Junior doctor no. 4, surgical specialty)

A resistance tactic of avoidance seems to be at play here. The interviewed junior doctors were happy with the way they were supervised in the everyday clinical setting, and since neither they nor their supervisors perceived any added value in using an assessment form, they simply omitted using it.  This can also be seen as a way of asserting autonomy, since their own, locally developed way of providing supervision in everyday clinical practice takes precedence over the strategically, externally imposed request to use formal, standardized assessment methods.

### Selection when arranging an assessment

Most of the junior doctors found it difficult to ask for formal assessment and therefore made use of different types of selection when asking to be assessed. Many had been met with rejection on the grounds of busyness when asking for formal assessment. Therefore, they would often approach senior doctors with whom they were on good terms in connection with assessment. One junior doctor from a medical ward said that she never even considered asking her main advisor to assess her because he always seemed busy and annoyed when she had asked him. In an interview she said:

“It is important that it’s someone you get along with. That’s actually why I chose [a certain doctor to assess her] […] because I think he’s a nice guy. I wouldn’t mind getting, like, criticism from him.” (Junior doctor no. 9, medical specialty)

Some of the junior doctors also recounted that they preferred asking doctors who were only slightly more experienced than they were for assessment. In an informal interview, one junior doctor from a medical specialty said that he preferred to be assessed by other young doctors because they gave better and more specific feedback than more experienced doctors who had forgotten what it was like to be a novice and gave more general advice (Junior doctor no. 1, medical specialty).

The junior doctors’ recounting of how they select colleagues to assess them suggests resistance from some senior doctors who are not eager to assess and refuse to do so on account of being busy – in turn making the junior doctors loathe to ask. It is also worth noting that many seem to feel more comfortable asking for assessment from someone close to their own educational level rather than more senior doctors, suggesting that hierarchy and authority within the ward also plays a role in connection with formative assessment.

### Ambiguous attitudes towards formative assessment methods

Overall, the ten interviewed junior doctors displayed an ambiguous attitude towards formative assessment methods, as demonstrated below.

#### The potential of formative assessment

On a positive note, the ten junior doctors all saw the potential of formative assessment in a number of different ways. In the interviews, some stated that they saw the assessment forms as reminders to learn and take responsibility for their own learning. One saw formative assessment as potentially giving rise to “reflection about myself. If I should maybe sometimes be better at asking ‘Would you take a look at so and so?’” (Junior doctor no. 5, paraclinical department). Others saw the assessment forms as a potential way to track progress or lack of progress, although none of the ten junior doctors of the study actually did so themselves. However, common to their statements were that they focused on the potential of formative assessment, rather than on what the junior doctors had actually experienced or made use of them-selves.

#### Different types of barriers to formative assessment

The junior doctors conveyed that the quality of the feedback given in connection with formative assessment varied considerably and did not always translate into learning. Many described that while some supervisors gave excellent feedback in general, others gave next to none or trivial feedback. One junior doctor from a medical specialty said of previous experience with a mini-CEX: “I got some feedback, but I didn’t think it was very original, no. But then it rarely is” (Junior doctor no. 10, medical specialty). Such experiences made the junior doctors less likely to seek out formative assessment methods again.

Most of the junior doctors voiced a number of different personal barriers to requesting a formative assessment. Some worried about disturbing busy colleagues. One junior doctor from a surgical specialty explained: “One reason is that you feel that you are using your colleague’s time. They’re busy” (Junior doctor no. 7, surgical specialty).

Some of the junior doctors described refraining from requesting formative assessment because of insecurity and awkwardness, in particular not wanting to make a faux pas in front of a colleague. One junior doctor felt it became increasingly difficult to ask for assessment the more experienced she became as a doctor:

“It’s easier the newer the doctor is, because at that point you’re like completely fresh and need some feedback, but now you feel that you’ve been here a while, so, therefore, it is a little bit […] well, toxic if you make any rookie mistakes.” (Junior doctor no. 6, surgical specialty)

Finally, some of the junior doctors associated the formative assessment methods and forms less with learning and more with bureaucracy. In addition, some saw the assessment forms as a type of tick box exercise made to meet documentation requirements rather than promote learning.

## Discussion

The purpose of this study was to explore how formative assessment methods are actually used in different clinical contexts. We found that challenges seemed to outweigh the perceived advantages of formal assessment methods. Central themes were a busy clinical workday, limited familiarity with the assessment methods, using other types of already integrated forms of teaching and learning, and selecting only sympathetic supervisors to assess them. Reasons for ambivalence towards the use of formal assessment methods were based on the varying quality of feedback, personal barriers, and associations with unnecessary bureaucracy. We have suggested that some of the approaches to formal assessment methods can be seen as resistance tactics in the shape of avoidance, deprioritizing, omission, asserting autonomy, and contesting the value of the assessment methods and forms.

The workplace-based assessment has been highlighted as a more reliable way to ensure that junior doctors acquire the necessary competencies 45.  Yet in spite of all of its (theoretical) merits, assessment as a method has proven difficult to implement in practice. Other qualitative studies have shown that the perception and use of work-place-based assessment is often less than optimal for a number of reasons: Assessment results are not considered proof of clinical competency, ^46^ attachment of learning value to assessment is limited,^47^^,^^48^ there is reluctance to provide constructive feedback to trainees,^49^ and there is an overall negative attitude towards assessment among both trainers and trainees.^28^^,^^47^^,^^50^^,^^51^

Our study adds the perspective that while formative assessment methods may in principle be beneficial for learning and patient safety, actually using assessment in the clinic can be a muddled, emotional, and inconvenient process where multiple agendas converge, and doctors may re-articulate, negotiate, and resist using the formative assessment methods as intended. This points to the importance of bearing in mind that technologies such as administrative, recording and reporting instruments or tools are often presented and perceived as neutral and objective means to achieve an end (in this case using assessment forms to assist learning and patient safety).^52^ However, they often also serve a disciplining purpose as formal regulatory mechanisms, in the case of assessment methods by imposing a fixed way of conducting supervision and thus formalizing activities and relationships that have traditionally been ad hoc and informal.^53^ Using everyday resistance theory as a lens in this study permit-ted insight into how the use of small, non-confrontational acts of resistance can be quite effective in delaying or hindering the implementation of formal assessment methods.

Our study also indicates that there is a limited familiarity with different assessment methods among both junior and senior doctors. This finding was somewhat surprising since all doctors in Denmark attend mandatory seminars which introduce workplace-based assessment. However, international studies have also shown there is a common tendency for clinical supervisors to lack knowledge of and skills in training methods and feedback.^54^ So perhaps it is not a question of more training in using the methods, but rather, as van der Vleuten & Verhoeven suggest, ensuring that the methods (and their underlying principles) are perceived as important and meaningful.^17^

This point seems especially critical seen in the light of our findings which indicates that junior doctors already feel their learning conditions are optimal without using the formative assessment methods. In our study, several junior doctors stated that the assessment methods seemed superfluous, since learning was already integrated in different ways in the clinical workday. Previous research has also pointed out that both junior and senior doctors are often skeptical about implementing assessment methods on the basis of the argument that `if it ain’t broke, don’t fix it’.^55^ Similarly, using formal assessment methods may also be at odds with the way clinicians like to teach. A previous study has shown that clinicians typically seize and create opportunities for learning spontaneously and opportunistically throughout the workday.^56^ Formative assessment might therefore be experienced as the opposite of this approach and a threat to autonomy, since it has to be planned and involves paperwork.

The current study underlines that workplace-based formative assessment seems to have what can be dubbed as an ‘image problem’ if it is perceived as having limited (learning) value. It needs to be regarded and experienced as meaningful and important to be used by both assessed and assessor. So far, few studies have addressed the issue of the educational impact of formative assessment on doctors’ learning and performance, and those that have show mixed results.^57^^,^^58^ More empirical research of this kind might be helpful and compelling in the implementation of assessment methods.

However, after more than 10 years of attempted implementation with mixed results, it might be time to reconsider whether workplace-based assessment in its present form is actually feasible. Is the lack of application merely a sign of expectable institutional inertia? Or is the format perhaps incompatible with the way the present-day clinic works? Although workplace-based assessment is, in theory, an advantageous approach to learning in the clinic, the clinic seems to resist, warranting more deliberation in this area.

### Study limitations

This study is based on short field observations and phone interviews, which means that there was only a limited time to observe and to build rapport with the informants. Furthermore, the observed assessment sessions may be seen as an ‘artificial set up’ in the sense that the junior doctors were observed on days when they were required to use a formative assessment method and observing them on an ordinary clinical workday might have painted a different picture. However, following the junior doctors on this specific day guaranteed that formative assessment would take place during observation. We have tried to take this into account in the analysis by not assuming to give a generalizable account, but rather to allow the complex nature of assessment to be explored.

There is a need for further observational studies on assessment methods, especially in terms of also exploring the faculty perspective. Furthermore, a longitudinal study in clinical settings where formative assessment methods are used successfully and routinely would provide richer research data on factors that promote formative assessment.

## Conclusions

This qualitative study explored how formative assessment methods are actually used and perceived in different clinical settings. The study shows that there are several barriers to the use of formative assessment methods in the clinical context, including subtle tactics of everyday resistance such as avoidance, deprioritizing, and contesting formative assessment methods.  The study also shows that junior doctors may have ambiguous attitudes towards the use of formal assessment methods as well as mixed experiences with their educational impact. The study emphasizes that while assessment methods may, in theory, be beneficial for learning and patient safety, carrying them out is not a neutral and context-independent exercise. Rather assessment takes place within complex contexts where it is affected by factors such as collegial and hierarchical relations, educational traditions, emotional issues, and subtle forms of resistance, including using assessment methods in creative ways. An important implication of this study is for the health care sector to address these issues in order for formative assessment methods to be properly implemented in the clinic.

### Acknowledgements

We would like to thank the junior doctors and their workplaces and colleagues for participating in this study. We would also like to thank the instructors of the guidance and supervision seminar for helping us to carry out the study.

### Conflict of Interest

The authors declare that they have no conflict of interest.
